# Modulation of the N13 component of the somatosensory evoked potentials in an experimental model of central sensitization in humans

**DOI:** 10.1038/s41598-021-00313-7

**Published:** 2021-10-21

**Authors:** A. Di Lionardo, G. Di Stefano, C. Leone, G. Di Pietro, E. Sgro, E. Malara, C. Cosentino, C. Mollica, A. J. Blockeel, O. Caspani, L. Garcia-Larrea, A. Mouraux, R. D. Treede, K. G. Phillips, M. Valeriani, Andrea Truini

**Affiliations:** 1 Department of Human Neuroscience, University Sapienza, Viale Università 30, 00185 Rome, Italy; 2 Department of Statistical Sciences, Sapienza University, Rome, Italy; 3 School of Physiology, Pharmacology and Neuroscience, University of Bristol, Bristol, UK; 4 Department of Neurophysiology, Mannheim Center for Translational Neurosciences (MCTN), Medical Faculty Mannheim, University of Heidelberg, Mannheim, Germany; 5 Lyon Neurosciences Center Research Unit Inserm U 1028, Pierre Wertheimer Hospital, Hospices Civils de Lyon, Lyon 1 University, Lyon, France; 6 Pain Center, Pierre Wertheimer Hospital, Hospices Civils de Lyon, Lyon 1 University, Lyon, France; 7 UCLouvain, Institute of Neuroscience (IoNS), Brussels, Belgium; 8 Neuroscience Next Generation Therapeutics, Eli Lilly and Company, Lilly Innovation Center, Cambridge, MA 02142 USA; 9 Department of Neuroscience, Headache Center, Bambino Gesù Children’s Hospital, Rome, Italy; 10 Center for Sensory-Motor Interaction, Aalborg University, Aalborg, Denmark

**Keywords:** Sensory processing, Neuropathic pain

## Abstract

The N13 component of somatosensory evoked potential (N13 SEP) represents the segmental response of dorsal horn neurons. In this neurophysiological study, we aimed to verify whether N13 SEP might reflect excitability changes of dorsal horn neurons during central sensitization. In 22 healthy participants, we investigated how central sensitization induced by application of topical capsaicin to the ulnar nerve territory of the hand dorsum modulated N13 SEP elicited by ulnar nerve stimulation. Using a double-blind placebo-controlled crossover design, we also tested whether pregabalin, an analgesic drug with proven efficacy on the dorsal horn, influenced capsaicin-induced N13 SEP modulation. Topical application of capsaicin produced an area of secondary mechanical hyperalgesia, a sign of central sensitization, and increased the N13 SEP amplitude but not the peripheral N9 nor the cortical N20-P25 amplitude. This increase in N13 SEP amplitude paralleled the mechanical hyperalgesia and persisted for 120 min. Pregabalin prevented the N13 SEP modulation associated with capsaicin-induced central sensitization, whereas capsaicin application still increased N13 SEP amplitude in the placebo treatment session. Our neurophysiological study showed that capsaicin application specifically modulates N13 SEP and that this modulation is prevented by pregabalin, thus suggesting that N13 SEP may reflect changes in dorsal horn excitability and represent a useful biomarker of central sensitization in human studies.

## Introduction

Central sensitization, a key mechanism contributing to several chronic pain conditions, is defined as an increase in the excitability of central nociceptive neurons (e.g. within the dorsal horn of the spinal cord)^1,2^. After a tissue injury, chemical mediators generate an area of primary hyperalgesia due to the peripheral sensitization of nociceptive fibres. Inputs into the spinal cord induced by a given stimulus consequently increase^3^. As a secondary effect, these increased inputs may induce sensitization of second order neurons in the central nervous system^4,5^ that manifests with a zone of secondary hyperalgesia, defined as the undamaged area surrounding the injury site with increased sensitivity to mechanical stimulation^5–9^.

Different experimental pain models have been devised to investigate the mechanisms underlying central sensitization in humans^10^. Peripheral injection and topical application of capsaicin have been found to intensively activate C and Aδ nociceptive nerve fibres, producing burning pain and pronounced flare response spreading beyond the area of primary hyperalgesia^10,11^.

Although capsaicin and other experimental pain models are commonly used to investigate the mechanisms underlying central sensitization and its association with chronic pain^12^, how to reliably quantify central sensitization within the dorsal horn of the human spinal cord is still an issue of controversy. Some studies have shown that the threshold of nociceptive flexion reflex (RIII) is reduced in human models of secondary hyperalgesia^13^, as well as in patients with chronic pain^12,13^. However, in the same studies the RIII amplitude remained unaffected, thus weakening the usefulness of RIII measurements as an objective biomarker of central sensitization^13–15^.

The N13 component of somatosensory evoked potentials (N13 SEP) recorded in anterior–posterior direction from the lower neck (Cv6) after upper limb stimulation is mediated by non-nociceptive Aβ fibres. This component, generated at the cervical dorsal horn^16^, probably reflects the segmental postsynaptic response of dorsal horn neurons in the lower cervical grey matter^17^, presumably neurons in laminae IV–V^18,19^. N13 SEPs might therefore constitute a neurophysiological measure sensitive to the excitability changes of dorsal horn neurons during central sensitization. In this neurophysiological study in healthy humans, we investigated if, and how capsaicin-induced central sensitization modulates the dorsal horn N13 SEPs. In addition, we tested whether pregabalin, a first-line treatment for neuropathic pain^20^ targeting voltage-gated calcium channels expressed in the dorsal horn and the brain^21^, is able to modulate capsaicin-induced N13 SEP sensitization.

## Methods

### Participants

We enrolled 10 healthy subjects for each experiment (overall 22 healthy subjects participated to this study; mean age 25.8 years, range 20–30 years; 11 males) without symptoms or signs of peripheral or central nervous system disorders or other medical conditions, any drug intake in the past two weeks, known or suspected allergic reactions or hypersensitivity to pregabalin or its components, dermatological disorders or skin lesions affecting the area of capsaicin application, jet lag, irregular working hours, sleep restrictions in the last week, or past drug abuse.

Written informed consent was obtained from all participants. This study was approved by the institutional review board of Sapienza, University of Rome (REF.CE 4789-2018) and performed according to the Declaration of Helsinki regarding the use of humans in experimental studies.

After informed consent, in a separate pilot session preceding at least one week the first experimental session, all subjects familiarised themselves with the technical procedures, including capsaicin application and electrical stimulation. During this experimental session, we assessed the consistency of secondary hyperalgesia and dynamic mechanical allodynia in the enrolled subjects.

### Experimental procedures

This study consisted of three distinct experiments. Experiment 1 was designed to verify whether central sensitization induced by application of a commercially available capsaicin cream (2.5% capsicum oleoresin corresponding to 0.1% active capsaicin associated with inactive ingredients) to the ulnar nerve territory of the right-hand dorsum affected N13 SEPs generated by ulnar nerve stimulation. In experiment 2, we investigated the time course of capsaicin-induced N13 SEP modulation. Experiment 3 was designed to verify whether the analgesic pregabalin, whose pharmacological target is expressed in the dorsal horn^21^, could prevent central sensitization-induced N13 SEP modulation; in a double-blind placebo-controlled crossover trial, we tested the effect of a single oral dose of pregabalin and placebo on the capsaicin-induced modulation of N13 SEPs.

Of 22 enrolled subjects, four participated in all the three experimental procedures. The three experiments were conducted at an interval of at least 3 months thus limiting any carry-over effects. Dorsal horn N13 SEP measures were analysed offline by two investigators who were unaware of the type of session (drug or placebo).

### Capsaicin-induced secondary hyperalgesia

**Figure 1:**
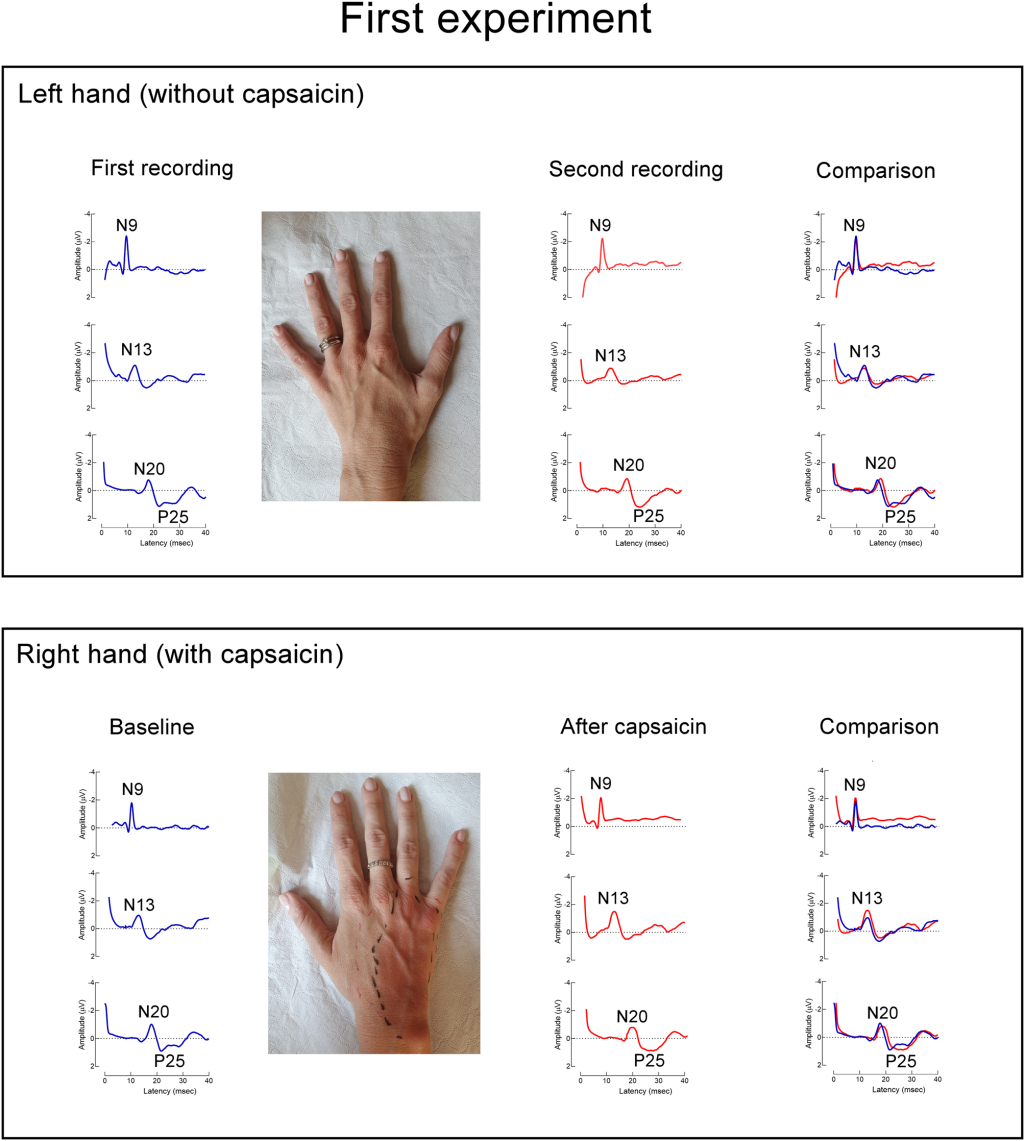
Experiment 1: Capsaicin-induced modulation of somatosensory evoked potentials (SEPs). Grand-average of SEP recordings. Topical capsaicin cream was applied on an area of about 15–18 cm^2^, covering almost the entire ulnar nerve territory of the hand dorsum in most subjects. This area corresponded to the area of primary hyperalgesia. N13 SEP amplitude increased after capsaicin application. The other SEP components (namely N9 and N20) did not change. The amplitude of SEP components after stimulation of the left hand did not change between the two recording sessions.

We applied topical capsaicin cream on the ulnar nerve territory of the right-hand dorsum, an area of approximately 15–18 cm^2^ covering almost the entire ulnar aspect of the hand dorsum (this area corresponded to the area of primary hyperalgesia). Capsaicin cream was applied for 40 (experiment 1 and 3) and 60 min (experiment 2), before being gently wiped off. We identified the site of primary hyperalgesia corresponding to the area of flare and burning pain, then we used a calibrated 128-mN pinprick probe (MRC Systems GmbH, Heidelberg, Germany) to map with six radial pinches the area of secondary pinprick hyperalgesia, defined as the area surrounding the flare zone with increased sensitivity to mechanical pinprick stimulation (Fig. 1). Participants received at least five pinprick stimuli for each radius, from outside towards the area of capsaicin application. The subjects were instructed to close their eyes and report when they feel a clear or erratic increase in pain or burning sensation. They were then asked to rate the average magnitude of pinprick secondary hyperalgesia using a numerical rating scale (NRS) ranging from 0 (no pinprick perception) to 100 (maximum pain imaginable). In the three experiments, mechanical pain sensitivity in the area of secondary hyperalgesia was assessed at each time point, before the SEP recording.

Given that in the pilot session an area of dynamic mechanical allodynia after capsaicin application was identified in 7 out of 10 participants only, we did not include this parameter in the experimental procedures and focused on secondary hyperalgesia to pinprick.

### Somatosensory evoked potential recording

Somatosensory evoked potentials (SEPs) were recorded after electrical stimulation of the ulnar nerve at the wrist (stimulus duration: 0.1 ms; stimulation frequency: 4 Hz; high-pass filter 2 Hz, low-pass filter 2 kHz; analysis time base: 50 ms; sampling rate: 16,384 Hz, System Plus Micromed). The cathode was placed over the distal ulnar nerve, 2 cm proximal to the wrist crease while the anode was placed on the wrist crease (the ulnar nerve stimulation was outside the area of primary hyperalgesia). Intensity was set at the threshold for evoking a muscle twitch in the muscles of the hand innervated by the ulnar nerve (10.4 ± 1.2 mA).

Three blocks of 500 trials were consecutively collected, superimposed (in order to evaluate reproducibility), and averaged. Muscle artefacts were avoided by making the patient as comfortable as possible. Subjects were instructed to lay down on a medical cot in a supine resting position. Automatic artefact rejection was used to eliminate occasional high-amplitude transients (> 100 μV).

To record and measure the different SEP components, we followed the International Federation of Clinical Neurophysiology guidelines^22^. The peripheral (N9) component was recorded with the surface electrode over the Erb point bilaterally, within the angle formed by the posterior border of the clavicular head of the sternocleidomastoid muscle and the clavicle, 2–3 cm above the clavicle. This montage served to monitor the peripheral input into the spinal cord. The dorsal horn N13 component was recorded with a posterior cervical spinal electrode over the sixth cervical spinous process, with an anterior cervical electrode as a reference on the skin surface of the supraglottic region on the midline. In each subject, N20 and P25 components were recorded with a parietal scalp electrode (CP3/CP4) contralateral to the stimulation, placed according to the 10–20 international system with an Fz reference. Impedance was kept below 5000 Ω. Epochs were averaged after automatic artefact rejection. In each subject less than 5% epochs were excluded. Peak amplitude of the different SEP components was manually extracted from the individual waveforms.

### Experiment 1

We enrolled 10 healthy participants (mean age 26.3 years, range 20–29 years; 4 males). Before capsaicin application, using the calibrated 128-mN pinprick probe, we quantified mechanical pain sensitivity in the right-hand dorsum with the 0–100 NRS (0: no pinprick perception; 100 maximum pain imaginable). After 40 min (this time interval was selected as the optimal time point due to robust secondary hyperalgesia in the majority of subjects during preliminary experiments) we mapped the area of secondary hyperalgesia and asked subjects to quantify mechanical pain sensitivity in the area of secondary hyperalgesia by using the 0–100 NRS.

We recorded SEPs in response to electrical stimulation of the ulnar nerve on both arms before and 40 min after capsaicin application to the ulnar nerve territory of the right-hand dorsum (after the mapping of secondary hyperalgesia area).

### Experiment 2

Ten healthy subjects (mean age 26.7, range 21–30 years; 4 males) participated in this experiment. We investigated the time course of capsaicin-induced N13 SEP modulation by recording dorsal horn N13 SEPs after stimulation of the ulnar nerve of the active side (treated with capsaicin) at multiple time intervals, namely before and 20, 40, 90, 120, 140, 160, and 180 min after capsaicin application to the ulnar nerve territory of the right-hand dorsum.

Participants were asked to rate the mechanical pain sensitivity in the area surrounding the capsaicin application, at baseline and at each time interval after capsaicin application, by using the 0–100 NRS (0: no pinprick perception; 100: maximum pain imaginable). In this experiment we did not map the area of secondary hyperalgesia.

### Experiment 3

In this experiment, we enrolled 10 healthy participants (mean age 26.3, range 21–30 years; 5 males). We recorded the N13 SEP after ulnar nerve stimulation of both arms before and 40 and 90 min after capsaicin application on the ulnar nerve territory of the right-hand dorsum. At each time point, participants were asked to rate the mechanical pain sensitivity, in the area surrounding the capsaicin application, by using a numerical rating scale (NRS) ranging from 0 (no pinprick perception) to 100 (maximum pain imaginable). The area of secondary hyperalgesia was mapped 40 min after the capsaicin application, immediately before the SEPs recording, by applying mechanical pinprick stimuli in the area surrounding the capsaicin-induced flare.

After the baseline recording, at the time of capsaicin application, subjects were randomised to receive a single oral dose of pregabalin 150 mg or placebo (a vitamin capsule) in a double-blind placebo-controlled crossover design. Placebo and pregabalin capsules were indistinguishable in terms of weight and size. Random numbers were assigned to each treatment condition. A minimum interval of 7 days was required before the subjects returned for their second session to minimise cross-over effects. The effectiveness of blinding was not directly assessed with specific measures.

### Statistical analysis

Descriptive summaries for the latencies and amplitudes of each SEP component (i.e. N9, N13, and N20-P25) for each treatment condition are presented as mean ± standard deviation (SD) (Table 1). Effect sizes are calculated as Cohen’s d.

**Table 1:** Experiment 1: Capsaicin induced modulation of somatosensory evoked potential components.

Side	Right ulnar nerve stimulation (active side)				Left ulnar nerve stimulation (control side)			
	Baseline	After capsaicin	p*	Effect size	Baseline	After capsaicin	p*	Effect size
N9 latency (ms)	9.7 ± 0.7	9.9 ± 0.8	0.69	0.26	9.7 ± 0.6	9.7 ± 0.7	0.83	0
N9 amplitude (µV)	2.1 ± 0.6	2.4 ± 0.7	0.58	0.46	2.4 ± 1.4	2.3 ± 1.6	0.9	0.06
N13 latency (ms)	13.1 ± 0.4	13.2 ± 0.2	0.94	0.31	13.1 ± 0.6	13.1 ± 0.7	0.97	0
N13 amplitude (µV)	0.96 ± 0.1	1.49 ± 0.4	0.001	1.8	1.1 ± 0.6	0.98 ± 0.3	0.53	0.25
N20 latency (ms)	18.9 ± 0.8	18.8 ± 1	0.86	0.11	18.7 ± 1	18.6 ± 1.1	0.99	0.09
N20-P25 amplitude (µV)	1.75 ± 0.5	1.8 ± 0.7	0.96	0.08	1.8 ± 0.7	2.1 ± 0.6	0.18	0.46

Data are expressed as mean ± SD.

Effect size are expressed as Cohen’s d.

*p values of post hoc Sidak’s multiple comparisons test (Two-way Repeated Measures ANOVA).

We used the Shapiro–Wilk test, recommended for the analysis of small samples, to assess data distribution^23^. No significant results were obtained from the Shapiro–Wilk test for the outcomes of interest, endorsing the plausibility of the normality assumption (0.07 < p < 0.89).

In experiment 1, paired t-test was used to assess NRS score differences before and after capsaicin application. Two-way repeated measures analysis of variances (ANOVA) and post hoc multiple comparison with Sidak’s correction was used to assess the effect of time (before and after capsaicin application) and treatment (capsaicin) and their interaction on latency and amplitude of SEP components.

Given that experiment 1 showed that peripheral N9 and cortical N20-P25 did not change, in experiments 2 and 3 the analysis was limited to N13 SEP amplitude.

In experiment 2 we performed one-way ANOVA with the Greenhouse–Geisser correction followed by Dunnett's multiple comparisons tests to assess N13 amplitude and NRS score variation across the different time points.

In experiment 3, we estimated a sample size of n = 10 subjects needed to detect a difference in N13 amplitude comparable to that observed in experiment 1, based on the effect size of interest (the N13 SEP amplitude change between active and control side, corresponding to 1.8; G-Power 3.0 α 0.05; β 0.90). We then performed a two-way repeated measures ANOVA on both arms including the Greenhouse–Geisser (GG) correction involving the effect of time, type of treatment and their interaction. Post hoc analysis of pairwise comparisons was performed using t-tests adjusted for multiple comparisons with Sidak’s correction.

We used the Pearson correlation coefficient to assess the correlation between changes (differences with baseline values) of the N13 SEP amplitude and the mechanical pain sensitivity in the 22 subjects included in this study at the 40-min time interval of the three experiments.

All the tests were two-sided and a *p*-value $\le$ 0.05 was considered statistically significant. All statistical analysis and plotting of data were performed in Prism 8.0 (GraphPad, CA, USA).

## Results

### Experiment 1

In each subject, capsaicin application to the ulnar nerve territory of the right-hand dorsum induced an area of primary hyperalgesia with flare, with a surrounding area of secondary hyperalgesia (10.6 ± 14.5 cm^2^) characterised by increased sensitivity to mechanical pinprick stimulation relative to the baseline assessment (baseline NRS: 33 ± 13.37; post-capsaicin NRS: 59 ± 15.24; paired t-test: p < 0.001).

The two-way repeated measure ANOVA revealed a significant interaction between time (before and after capsaicin) and type of treatment (capsaicin, control site) for the N13 SEP amplitude (p < 0.02) (Supplementary Table 1). Post hoc analysis showed a significant increase of the N13 SEP amplitude after treatment on the capsaicin-treated side (p < 0.005; Cohen’s d effect size 1.8). No significant results were found for the other SEPs variables (peripheral N9, cortical N20-P25, Table 1), (Fig. 1; Supplementary Fig. 1; Supplementary Tables 1, 2).

### Experiment 2

**Figure 2:**
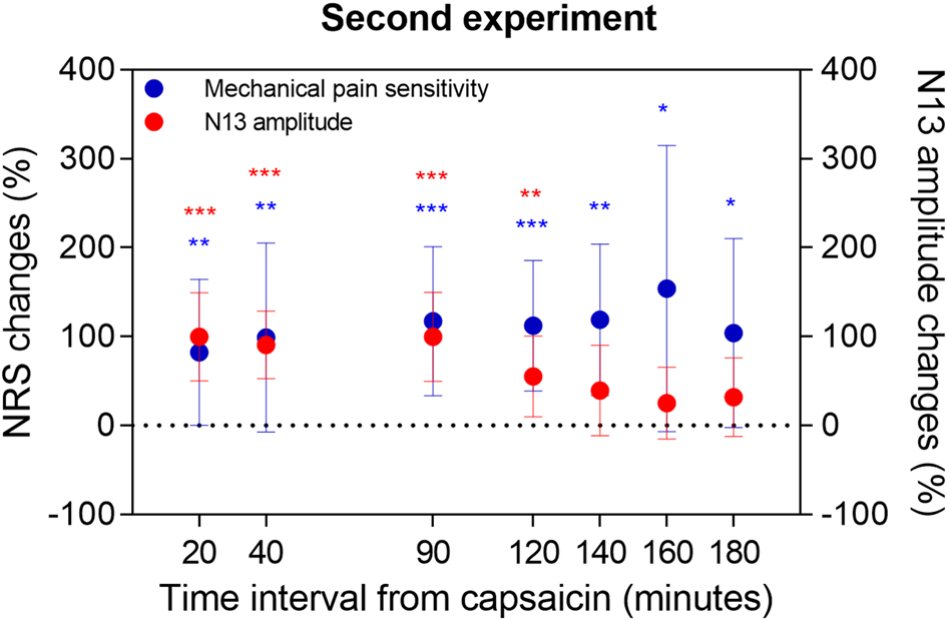
Experiment 2: Time course of capsaicin-induced modulation of N13 somatosensory evoked potentials (SEPs). Red dots: N13 amplitude changes (at each interval N13 SEP amplitude was normalized to baseline amplitude); blue dots: secondary hyperalgesia rating (at each interval the magnitude of mechanical pain sensitivity was normalized to baseline magnitude as assessed by a 0–100 numerical rating scale). Dots represent mean and standard deviations. Asterisks indicate significance vs. baseline (Dunnett’s corrected p-value), *p < 0.05, **p < 0.01, ***p < 0.001.

The time course analyses showed that capsaicin application increased the amplitude of the N13 SEP for up to 120 min (Geisser-Greenhouse's epsilon = 0.4038, p < 0.01), while secondary mechanical hyperalgesia, as quantified by the increase in pain sensitivity relative to baseline, had a longer duration of up to 180 min (Geisser-Greenhouse's epsilon = 0.3629, p < 0.01), (Fig. 2; pairwise comparisons and effect sizes are detailed in Supplementary Table 3).

### Experiment 3

**Figure 3:**
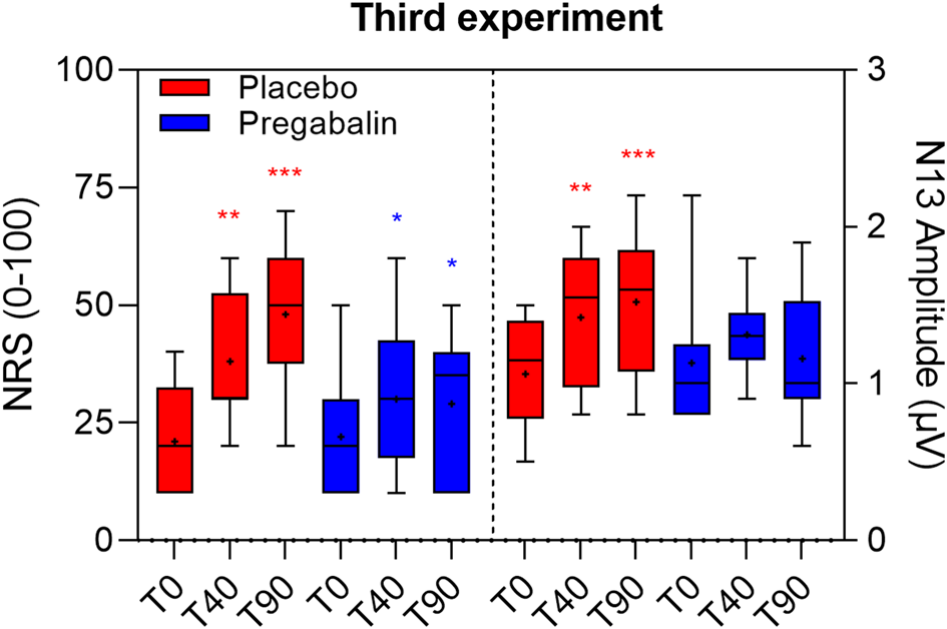
Experiment 3: Crossover trial assessing how pregabalin prevents capsaicin-induced modulation of N13 somatosensory evoked potentials (SEPs) and Secondary Hyperalgesia. Boxplots of the N13 amplitudes and magnitude of secondary hyperalgesia (NRS 0–100) at different time points (placebo in red, pregabalin in blue). Pregabalin prevented capsaicin induced facilitation of N13 and attenuated that of hyperalgesia ratings. Black lines represent medians, crosses represent means. Asterisks indicate significance vs. baseline (Sidak-corrected p-value), *p < 0.05, **p < 0.01, ***p < 0.001.

Comparisons between pregabalin treatment and placebo are shown in Fig. 3 for NRS of secondary hyperalgesia and N13 amplitude. For individual subject data, see Supplementary Fig. 2.

When we analysed magnitude changes of secondary hyperalgesia, we found that the two-way repeated measure ANOVA showed a significant effect of time (GG epsilon = 0.5898, p < 0.001) and time × treatment interaction (GG epsilon = 0.9863, p = 0.0119) (Supplementary Table 4). The post hoc pairwise analysis identified significant increases above baseline in the placebo session during the entire follow-up. In the pregabalin session the trend of the NRS values was also significant but lower in magnitude; an increment occurred after the 40-min measurement session, followed by a slight negative tendency (Supplementary Table 5). The pairwise comparison analysis between treatment sessions showed that at 90 min after capsaicin application the NRS values were significantly higher in the placebo than in the pregabalin session (p < 0.05; Supplementary Table 5).

When we analysed changes of the N13 SEP amplitude we found that the two-way repeated measure ANOVA did not show any significant changes of the N13 SEP in the control side (Supplementary Table 6). Conversely, we found a significant effect of time (GG epsilon = 0.8288, p = 0.009) and interaction with the type of treatment (GG epsilon = 0.8016, p = 0.0214) for the capsaicin-treated side, indicating that the variation of the outcome follows a significantly different trend over time depending on the treatment session (Supplementary Table 5). Specifically, the post hoc pairwise analysis showed that, in the placebo session, the N13 SEP amplitude significantly increased at 40 and 90 min. Conversely, N13 SEP amplitude did not differ across the three recordings in the pregabalin treatment session (Fig. 3; Supplementary Fig. 2).

At the end of the experimental procedures three subjects reported somnolence (all in the pregabalin treatment session). No other adverse events were reported.

The correlation analysis in the 22 subjects included in this study, did not show any significant correlation between the N13 SEP amplitude changes and the magnitude changes of mechanical pain sensitivity before and 40 min after capsaicin application (p = 0.6; r = − 0.14).

## Discussion

In this neurophysiological study in healthy humans, we show that central sensitization induced by topical capsaicin was associated with an increase in the amplitude of the dorsal horn N13 SEP. Furthermore, we showed that pregabalin, a drug with proven efficacy on dorsal horn neurons, prevented the N13 SEP modulation associated with capsaicin-induced central sensitization. These findings suggest that N13 SEP reflects changes in dorsal horn excitability and might represent a biomarker of central sensitization in humans.

To investigate the effect of central sensitization on the N13 SEP, we used topical capsaicin, a safe and easy-to-use experimental model to induce central sensitization and secondary hyperalgesia, which avoids the often very strong pain induced by other routes of administration such as intradermal injection^12^. Further experimental pain models could also be potentially used to investigate N13 SEP changes during central sensitization, such as high-frequency electrical stimulation of the skin, which also elicits robust secondary hyperalgesia, but potentially has a longer and more stable time course than topical capsaicin^24–26^.

In our experiments, we analysed N13 SEP amplitude changes at multiple time points after capsaicin application which spanned a period of stable secondary hyperalgesia which persisted for up to 180 min. Since all subjects reported robust secondary hyperalgesia between 30 and 60 min after capsaicin application in the experimental session, we used a 40-min time interval in experiments 1 and 3 to assess the capsaicin-induced N13 SEP modulation. In experiment 3, we investigated whether pregabalin also prevented capsaicin-induced N13 SEP modulation at 90 min, given that 60–90 min is the time of maximum observed pregabalin plasma concentration^27^.

Each experiment in our study (including the placebo treatment session in experiment 3) concordantly demonstrated that topical application of capsaicin cream to the ulnar nerve territory of the hand dorsum produced a mild, though reproducible, increase in the N13 SEP amplitude after electrical stimulation of the ulnar nerve. This increase was associated with an elevated pinprick response in the area of secondary hyperalgesia, which is also a function of central sensitization. The N13 SEP, which is mediated by non-nociceptive Aβ fibres, is generated by segmental postsynaptic responses of dorsal horn neurons at the level of the cervical spinal cord^18,28^. N13 SEP characteristics in humans are very similar to those of the N1 spinal potential in animal models, reflecting postsynaptic neuronal responses to inputs conveyed by group I and II peripheral afferent fibres in laminae IV and V of the dorsal horn^28–30^. We therefore conjecture that our data indicate that the increase of the N13 SEP after topical capsaicin reflects dorsal horn excitability changes underlying central sensitization.

Previous studies have shown that capsaicin induces an input amplification in nociceptive specific neurons as well as wide-dynamic range (WDR) neurons with convergent tactile and nociceptive inputs; low-threshold Aß fibre-related neurons with purely tactile inputs are not facilitated^31^. Since N13 SEP is elicited by Aß fibre stimulation, its amplitude increase might therefore reflect an excitability change of WDR neurons.

Peripheral N9 SEP and cortical N20-P25 SEP did not parallel N13 SEP amplitude modifications across the three time points of the experiment. The cortical SEP components are generated in the primary somatosensory cortex by large myelinated fibres whose collaterals activate dorsal horn neurons generating N13 SEP^22^. These findings lend further evidence that the N13 SEP may reflect dorsal horn excitability.

The experiment 2 of our study showed that the capsaicin-induced N13 SEP modulation persisted for approximately 2 h, consistent with long-lasting, yet reversible, potentiation of spinal nociceptive pathways^24^. This long-lasting modulation is presumably compatible with post-translational changes associated with the early phase of long-term potentiation underlying central sensitization^25^. Conversely, secondary hyperalgesia, quantified by the increase in mechanical pain sensitivity in comparison to baseline assessment, had a longer duration. This difference may be compatible with a supra-spinal contribution to this long-lasting increased mechanical pain sensitivity. This interpretation is in line with previous studies showing decay of dorsal horn sensitization after 2 h^24^, with longer-lasting metabolic changes in supraspinal structures^32^.

In experiment 3, we found that pregabalin reduced the secondary hyperalgesia magnitude in comparison with the placebo. In the pregabalin session the NRS increase was lower than that in the placebo session and this increase had a slight negative tendency at 90 min. The effect of pregabalin we found is consistent with previous studies showing that gabapentinoids reduce hyperalgesic responses associated with capsaicin-induced central sensitization^33,34^. Notably, we found that pregabalin completely prevented the N13 SEP modulation associated with capsaicin-induced central sensitization, thus the effects on spinal signalling were stronger than those on perception. Pregabalin inhibits voltage-gated calcium channel activity at the presynaptic level, thus reducing the synaptic release of neurotransmitters and activation of postsynaptic neurons^21^. Although we cannot exclude that pregabalin might affect the N13-SEP through supraspinal pharmacological effects, we consider that the most plausible mechanism is a reduction of the central sensitization induced by capsaicin at the level of the dorsal horn^21^.

Our data therefore introduce the possibility of using the N13 SEP as a biomarker to investigate how drugs affect central sensitization in the human spinal cord. European Medicines Agency guidelines on the clinical development of medicinal products intended for the treatment of pain explicitly indicate that objective biomarkers might improve the development of drugs for chronic pain (https://www.ema.europa.eu/en/documents/scientific-guideline/guideline-clinical-development-medicinal-products-intended-treatment-pain-first-version_en.pdf). Our data indicate that the dorsal horn mediated-N13 SEP might be used to detect central sensitization in human clinical trials and ultimately demonstrate its modulation by novel analgesics; large group comparison may provide useful data on dorsal horn excitability changes during central sensitization. Since many analgesic drugs have failed when tested in clinical populations^35^, the use of N13 SEPs in early phase pharmacological trials might hasten the identification and selection of promising drug candidates for chronic pain, thereby helping to increase the likelihood of successful translation from the preclinical to clinical settings and thereby reducing the high costs associated with their development.

Our data showing that topical application of capsaicin modulates the N13 SEP amplitude argues against two previous studies showing that motor task and experimental capsaicin-induced pain do not significantly affect N13 SEP^36,37^; these contrasting results probably reflect the different experimental designs. In these two studies, capsaicin was used at low concentration, applied on the glabrous skin of the hand, and its effect assessed early (20 min); furthermore, these two studies do not report whether secondary hyperalgesia developed or not.

Alternative neurophysiological biomarkers might be used to test central sensitization. Previous studies showed that the nociceptive flexion reflex excitability is increased in patients with chronic pain conditions, presumably associated with central sensitization^14,15^. However, these studies did not consistently report a consensual amplitude increase of the nociceptive flexion reflex^13–15^. Furthermore, nociceptive flexion reflex is also affected by ventral horn motoneuron excitability, and, contrary to N13 recordings, needs to employ noxious stimuli which limits patient acceptability. Together, these limitations hamper the usefulness of nociceptive flexion reflex measurements for assessing central sensitization. Previous studies have shown increased amplitude of pinprick-evoked potentials after stimulation of the area of secondary hyperalgesia in healthy humans^38–42^. The increased vertex complex of pinprick-evoked potentials may reflect an increased cortical response to A-fibre mechano-nociceptive inputs due to capsaicin-induced central sensitization. Our study, however, suggests that caudal N13 SEPs generated by the dorsal horn may provide a direct measure of increased dorsal horn excitability during central sensitization. The dorsal horn N13 SEP is mediated by non-nociceptive Aβ fibres, which is a crucial advantage in clinical settings since stimulation is set at non-painful levels, hence ensuring subjects’ compliance. A dorsal horn SEP component can be recorded after stimulation of lower limbs (N22 SEP), thus allowing the investigation of central sensitization across multiple sites^43^.

### Limitations

We did not find any significant correlation between the N13 SEP amplitude and the magnitude of secondary hyperalgesia. This finding might be due to a poor amplitude resolution (signal-to-noise ratio) of N13 SEP recordings, which might prevent the possibility to detect minor excitability changes, still able to produce perceptual changes.

Admittedly, we cannot exclude that attention might concur in the N13 SEP amplitude changes we found. Previous studies showed that attention modulates dorsal horn mediated nociceptive responses such as the nociceptive flexion reflex^44^.

In our study, we stimulated the ulnar nerve to elicit N13 SEPs because the topical capsaicin model works best on hairy skin^12^, though this appears to yield lower amplitude SEP components than the more commonly used median nerve stimulation^22^. The relatively low amplitude of ulnar-nerve-mediated N13 SEPs and the mild amplitude increase after capsaicin application probably prevent the assessment of capsaicin-induced modulation at single subject level. This limitation may hamper the usefulness of our findings in the search for objective evidence of central sensitization in individual patients with neuropathic pain, but at a group level such changes have already been reported for patients with painful cervical radiculopathy^45^. N13 SEP investigation might still be useful to assess drug effects on central sensitization in pharmacological trials since drug effects are assessed using statistical group comparisons in these studies.

Although in our three experiments including 22 participants we found that the capsaicin-induced effect on the N13 SEP modulation is relatively reproducible, further studies, including larger sample of healthy participants, are needed to further support a regular use of N13 SEP as a biomarker of central sensitization.

## Conclusions

The dorsal horn N13 component of SEPs reflects dorsal horn excitability changes, thus raising the possibility that this spinal SEP component might be used as a biomarker to investigate central sensitization in humans. N13 SEPs may therefore represent a useful tool to detect central sensitization and to test the effects of drugs on pain in pharmacological trials involving humans.

## Supplementary Information

Supplementary Figure 1.

Supplementary Figure 2.

Supplementary Tables.

## Data Availability

The datasets generated during and/or analysed during the current study are available from the corresponding author on reasonable request.
