# Supplementary material for: Modulation of the N13 component of the somatosensory evoked potentials in an experimental model of central sensitization in humans
Source: Sci Rep. 2021 Oct 21;11:20838. doi: 10.1038/s41598-021-00313-7 (PMC8531029; doi:10.1038/s41598-021-00313-7)
Supplement: Supplementary file 1 — Supplementary Figure 1. [file 41598_2021_313_MOESM1_ESM.docx]

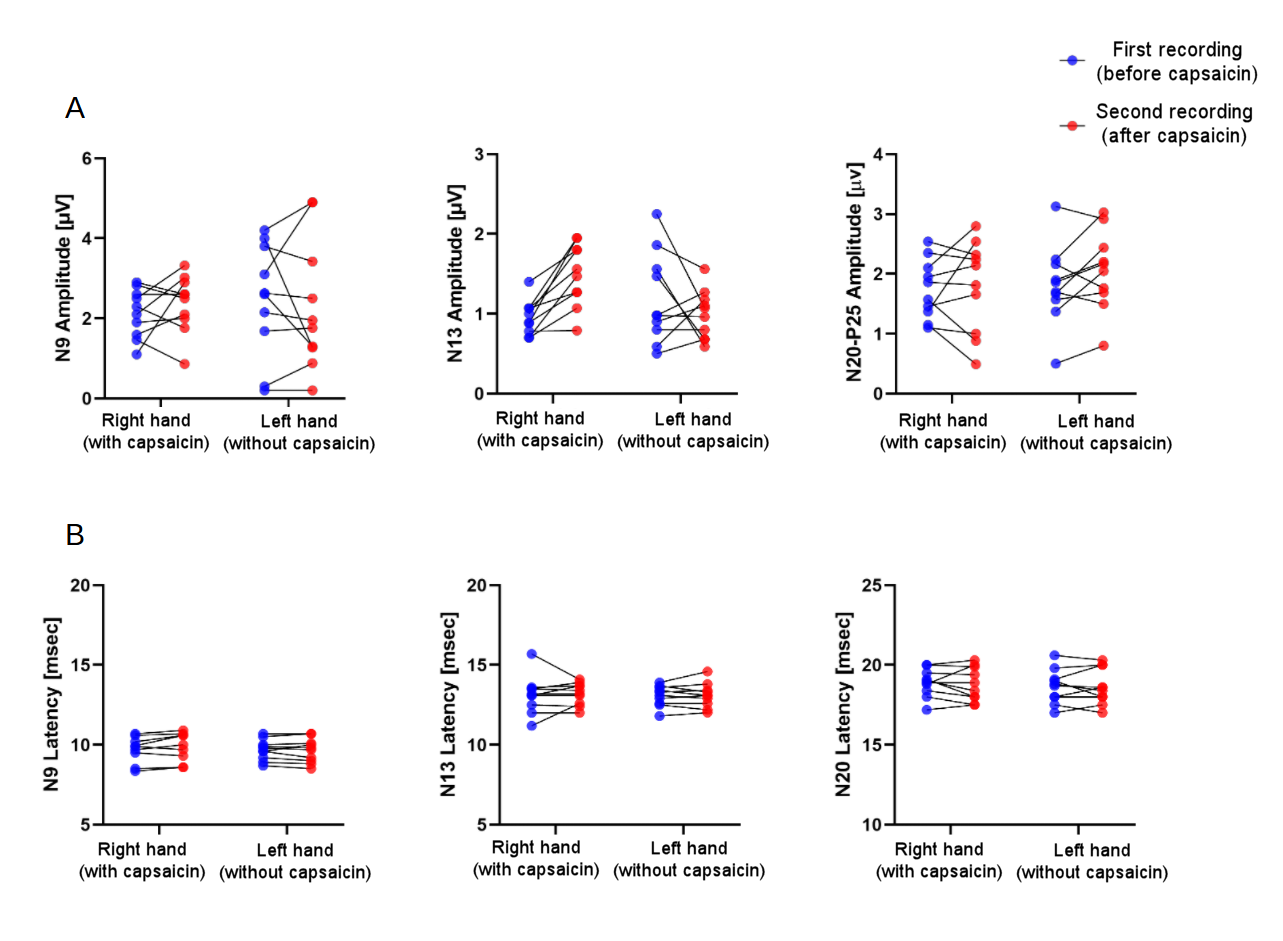


**Supplementary figure 1**. SEP variable changes (A: amplitudes; B: latencies) between the first and second recording (before and after capsaicin) in the ten subjects included in experiment 1.
