# Supplementary material for: Modulation of the N13 component of the somatosensory evoked potentials in an experimental model of central sensitization in humans
Source: Sci Rep. 2021 Oct 21;11:20838. doi: 10.1038/s41598-021-00313-7 (PMC8531029; doi:10.1038/s41598-021-00313-7)
Supplement: Supplementary file 2 — Supplementary Figure 2. [file 41598_2021_313_MOESM2_ESM.docx]

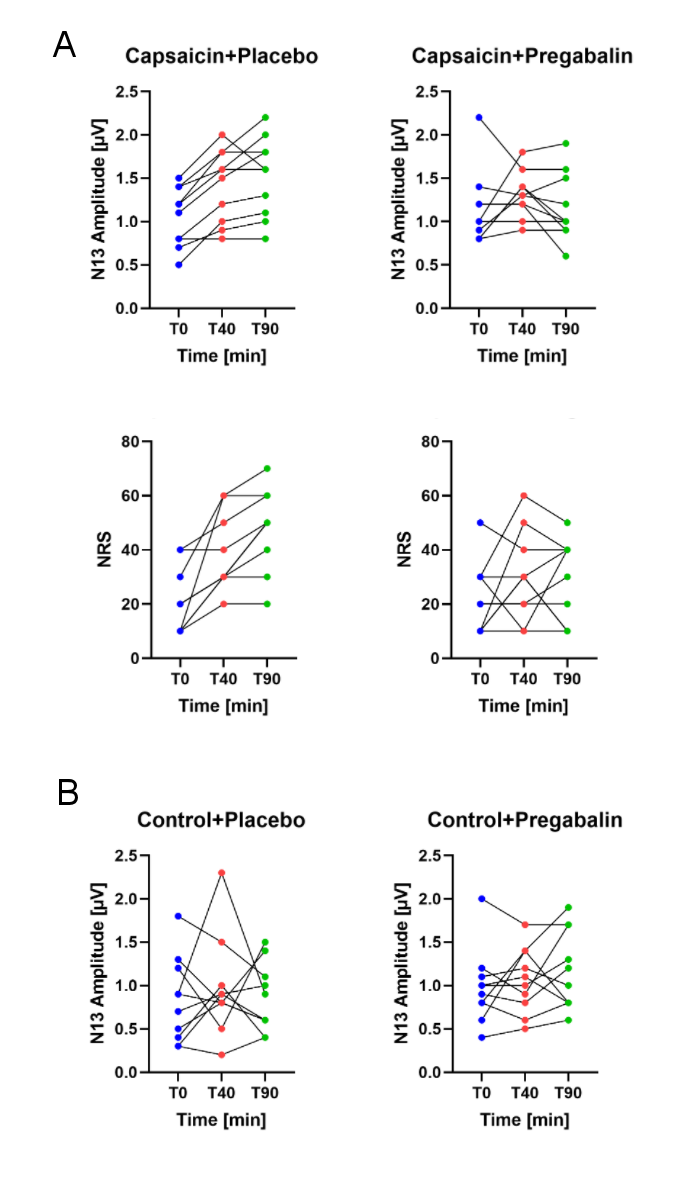


**Supplementary figure 2.** N13 SEP amplitude and pinprick perception changes across the three time points in the active side (A) and in the control side (B), in the experiment 3. N13 SEP was recorded after stimulation of both arms before and 40 and 90 minutes after capsaicin application to the right hand dorsum. In the left hand without capsaicin application (control), the pinprick perception was not evaluated.
