# Supplementary material for: Modulation of the N13 component of the somatosensory evoked potentials in an experimental model of central sensitization in humans
Source: Sci Rep. 2021 Oct 21;11:20838. doi: 10.1038/s41598-021-00313-7 (PMC8531029; doi:10.1038/s41598-021-00313-7)
Supplement: Supplementary file 3 — Supplementary Tables. [file 41598_2021_313_MOESM3_ESM.docx]

**Table 1: F-test p-values of the two-way repeated measures ANOVA for the SEP amplitude components in experiment 1.**

|  | N9 Amplitude  (µV) | N13 Amplitude  (µV) | N20 Amplitude  (µV) |
| --- | --- | --- | --- |
| Factor | p-value | p-value | p-value |
| Time | 0.8826 | 0.1225 | 0.1639 |
| Treatment | 0.7722 | 0.3177 | 0.1531 |
| Time* Treatment | 0.3065 | 0.0108 | 0.4425 |

**Table 2: F-test p-values of the two-way repeated measures ANOVA for SEP latency components in experiment 1.**

|  | N9 Latency  (ms) | N13 Latency  (ms) | N20 Latency  (ms) |
| --- | --- | --- | --- |
| Factor | p-value | p-value | p-value |
| Time | 0.4265 | 0.6793 | 0.7449 |
| Treatment | 0.5260 | 0.5329 | 0.1893 |
| Time* Treatment | 0.4636 | 0.8747 | 0.6500 |

**Table 3: Effect sizes and adjusted p-values of experiment 2**

Cohen’s d (effect size) and adjusted p-values for the pairwise comparisons of mechanical pain sensitivity and N13 amplitude values.

|  | Mechanical pain sensitivity  (Numerical rating scale) | | N13 Amplitude  (µV) | |
| --- | --- | --- | --- | --- |
| Contrasts | Effect size | Dunnett’s-corrected p-value | Effect size | Dunnett’s-corrected p-value |
| Baseline vs 20 mins | 1.3 | 0.0067 | 2.7 | <0.0001 |
| Baseline vs 40 mins | 1.6 | 0.0057 | 2 | <0.0001 |
| Baseline vs 90 mins | 2.1 | 0.0005 | 2.1 | 0.0001 |
| Baseline vs 120 mins | 2 | 0.0004 | 1.1 | 0.0068 |
| Baseline vs 140 mins | 1.8 | 0.0048 | 0.78 | 0.2154 |
| Baseline vs 160 mins | 1.6 | 0.0323 | 0.55 | 0.3241 |
| Baseline vs 180 mins | 1.3 | 0.0443 | 0.6 | 0.2300 |

**Table 4: Two-way repeated measures ANOVA of experiment 3**

F-test p-values of the two-way repeated measures ANOVA of mechanical pain sensitivity and N13 SEP amplitude values of the active side.

| Mechanical pain sensitivity  (Numerical rating scale) | | N13 Amplitude  (µV) | |
| --- | --- | --- | --- |
| Factor | p-value | Factor | p-value |
| Time | <0.001 | Time | 0.009 |
| Treatment | 0.070 | Treatment | 0.234 |
| Time* Treatment | 0.012 | Time* Treatment | 0.021 |

**Table 5: effect sizes and adjusted p-values of experiment 3**

Cohen’s d (effect size) and adjusted p-values for the pairwise comparisons of the mechanical pain sensitivity and N13 amplitude values of the active side.

|  | Mechanical pain sensitivity  (Numerical rating scale) | | N13 Amplitude  (µV) | |
| --- | --- | --- | --- | --- |
| Contrasts | Effect size | Sidak-corrected  p-value | Effect size | Sidak-corrected  p-value |
| **PLACEBO** |  |  |  |  |
| Baseline vs 40 mins | 1.3 | 0.005 | 0.95 | 0.0151 |
| Baseline vs 90 mins | 2 | <0.001 | 1.1 | 0.0014 |
| 40 mins vs 90 mins | 0.7 | 0.0113 | 0.23 | 0.8019 |
| **PREGABALIN** |  |  |  |  |
| Baseline vs 40 mins | 0.54 | 0.0151 | 0.48 | 0.3801 |
| Baseline vs 90 mins | 0.49 | 0.0148 | 0.07 | 0.9928 |
| 40 mins vs 90 mins | 0.06 | 0.8020 | 0.45 | 0.5355 |
| **PREGABALIN vs PLACEBO** | |  |  |  |
| Baseline | 0.07 | 0.9973 | 0.18 | 0.9365 |
| 40 mins | 0.5 | 0.5865 | 0.31 | 0.8193 |
| 90 mins | 1.26 | 0.0328 | 0.85 | 0.0482 |

**Table 6: Two-way repeated measures ANOVA of experiment 3**

F-test p-values of the two-way repeated measures ANOVA of N13 SEP amplitude values of the control side.

| N13 Amplitude  (µV) | |
| --- | --- |
| Factor | p-value |
| Time | 0.6134 |
| Treatment | 0.9786 |
| Time* Treatment | 0.3243 |
